# Genome-Wide Association Study and Transcriptome Analysis Reveal Alkaline Stress-Responsive Genes in Bread Wheat (*Triticum aestivum* L.)

**DOI:** 10.3390/ijms26178659

**Published:** 2025-09-05

**Authors:** Xuelian Sun, Xin Kang, Jiayan Wang, Xiaoyan He, Wenxing Liu, Dengan Xu, Xuehuan Dai, Wujun Ma, Jianbin Zeng

**Affiliations:** College of Agronomy, Qingdao Agricultural University, Qingdao 266109, China; 20232101043@stu.qau.edu.cn (X.S.); kangxin20041001@163.com (X.K.); wjy@stu.qau.edu.cn (J.W.); hexiaoyan@qau.edu.cn (X.H.); liuwx@qau.edu.cn (W.L.); xudengan@qau.edu.cn (D.X.); 202101034@qau.edu.cn (X.D.)

**Keywords:** *Triticum aestivum*, GWAS, RNA-seq, alkaline stress, candidate genes

## Abstract

Alkaline stress, driven by high pH and carbonate accumulation, results in severe physiological damage in plants. While the molecular mechanisms underlying alkaline tolerance have been partially elucidated in many crops, they remain largely unexplored in wheat. We hypothesize that alkaline stress tolerance in wheat is genotype-dependent. This study employed an integrated multi-omics approach to assess alkaline stress responses, combining genome-wide association study (GWAS) and RNA-seq analyses. Systematic phenotyping revealed severe alkaline stress-induced root architecture remodeling—with 57% and 73% length reductions after 1- and 3-day treatments, respectively—across 258 accessions. Analysis of the GWAS results identified nine significant alkaline tolerance QTLs on chromosomes 1A, 3B, 3D, 4A, and 5B, along with 285 associated candidate genes. Using contrasting genotypes—Dingxi 38 (tolerant) and TDP.D-27 (sensitive)—as experimental materials, physiological analyses demonstrated that root elongation was less inhibited in Dingxi 38 under alkaline stress compared to TDP.D-27, with superior root integrity observed in the tolerant genotype. Concurrently, Dingxi 38 exhibited enhanced reactive oxygen species (ROS) scavenging capacity. Subsequent RNA-seq analysis identified differentially expressed genes (DEGs) involved in ion homeostasis, oxidative defense, and cell wall remodeling. Integrated GWAS and RNA-seq analyses allowed for the identification of seven high-confidence candidate genes, including transcription factors (MYB38, bHLH148), metabolic regulators (ATP-PFK3), and transporters (OCT7), elucidating a mechanistic basis for adaptation to alkaline conditions. These findings advance our understanding of alkaline tolerance in wheat and provide candidate targets for molecular breeding of saline- and alkaline-tolerant crops.

## 1. Introduction

Saline and alkaline soils represent distinct but often co-occurring constraints in global agricultural ecosystems, predominantly distributed across the arid and semi-arid regions of Asia, Africa, Australia, and South America [1]. Saline soils are characterized by high concentrations of soluble salts (e.g., NaCl, Na_2_SO_4_), whereas alkaline (sodic) soils exhibit high levels of exchangeable sodium (Na^+^) and elevated pH, leading to poor soil structure and reduced permeability [2]. According to the Food and Agriculture Organization of the United Nations (FAO), over 900 million hectares of land worldwide are affected by varying degrees of salinization or alkalization, among which alkaline soils account for over 20%, while the remainder are primarily saline or saline–alkaline mixed soils [2]. In China, the total area of salt-affected land (including saline, alkaline, and saline–alkaline mixed soils) is approximately 99.13 million hectares—primarily concentrated in the three northeastern provinces, the inland northwest regions, and the Huang-Huai-Hai Plain—posing a substantial challenge to agricultural sustainability and food security [3]. This situation is further exacerbated by climate change and improper irrigation practices, leading to the continuous expansion of both saline and alkaline areas.

While salt and alkaline stresses commonly occur together in nature, they act through unique pathways and evoke varied reactions in plants. Salt stress, driven primarily by neutral salts such as NaCl, induces osmotic stress and ion toxicity [4,5,6]. In contrast, alkaline stress, typically caused by carbonate accumulation (e.g., Na_2_CO_3_ and NaHCO_3_), not only triggers osmotic and ionic imbalances but also subjects plants to high pH stress, nutrient deficiencies (e.g., impaired uptake of Fe, P, and Ca), and cellular structural damage [7,8,9,10]. For instance, cotton adapts to osmotic stress primarily through the accumulation of organic osmolytes (e.g., organic acids, amino acids, and sugars) under salt stress; in contrast, under alkaline stress, osmoregulation is mainly mediated by the uptake and compartmentalization of inorganic ions [11]. Studies have suggested that alkaline stress typically causes more severe physiological harm to plants compared to salt stress. For various crops, alkaline stress strongly hinders seed sprouting, root development, and biomass accumulation while disrupting photosynthesis, hormone metabolism, and cell wall integrity, ultimately leading to yield reduction and quality degradation [12,13].

Recent advances in molecular biology and high-throughput technologies have deepened our understanding of plant responses to alkaline stress. Studies have revealed that plants adapt to alkaline conditions through multifaceted strategies, including ion transport regulation (e.g., H^+^-ATPase and Na^+^/H^+^ antiporters), hormonal signaling (e.g., ABA and JA), antioxidant defense systems (e.g., POD and SOD), and transcriptional reprogramming (e.g., NAC, bZIP, and MYB transcription factors) [10,14]. For instance, it has been shown that AT1 (*Alkali tolerance 1*)—the major alkaline tolerance gene in sorghum—functions by modulating the phosphorylation of aquaporins, particularly PIP2s [15]. These aquaporins facilitate the efflux of excess hydrogen peroxide (H_2_O_2_) from cells, thereby lowering intracellular H_2_O_2_ levels and mitigating oxidative damage. In cotton, the *GhGLDH35A* gene regulates alkaline tolerance by modulating the biosynthesis of ascorbic acid and maintaining intracellular reactive oxygen species (ROS) homeostasis [16]. In rice, the *OsSAP6* gene interacts with *OsPK5* gene to positively regulate soda saline–alkaline tolerance through ROS homeostasis [17]. Additionally, *ZmNSA1* regulates Na^+^ homeostasis by promoting root Na^+^ efflux through the Ca^2+^-dependent degradation of *ZmNSA1*, which up-regulates PM-H^+^-ATPases (*MHA2/MHA4*) and enhances SOS1-mediated Na^+^/H^+^ antiport activity [18].

Wheat (*Triticum aestivum* L.), a staple crop that is vital to global food security, suffers an estimated 15% annual yield loss due to saline–alkaline stress. Integrated multi-omics analyses, combining QTL mapping, transcriptomics (RNA-seq), proteomics, and metabolomics, have enabled the identification of multiple alkaline-responsive genes in wheat, including *TaCCD1*, *TaSAUR215*, *TaWRKY55*, and *TaPLATZ2* [19,20]. Nevertheless, research on alkaline stress remains limited when compared to salt stress, particularly regarding the functional characterization of key regulatory genes and integrated stress response mechanisms. In this study, we employed an integrated multi-omics approach combining GWAS and RNA-seq to address two key hypotheses: (1) Alkaline stress triggers distinct molecular responses in wheat compared to salt stress, involving specific genetic loci and metabolic pathways; and (2) candidate genes regulating these responses can be functionally validated as determinants of alkaline tolerance. To test these hypotheses, we aimed to (i) identify alkaline-responsive QTLs and co-expression networks through GWAS and transcriptomics; (ii) prioritize candidate genes by correlating genomic loci with differential expression and stress phenotypes; and (iii) propose a mechanistic model of alkaline tolerance for validation in future studies.

## 2. Results

### 2.1. Effects of Alkaline Salt Stress on Wheat Phenotype

Phenotyping of 258 wheat accessions revealed alkaline stress-induced modulation of primary root growth kinetics, with comparative measurements under control and stress conditions (pH 9.5) at 24- and 72 h intervals generating four quantitative trait profiles (Supplementary Table S1). The results showed that root length (RL) under control was significantly higher than that under treatment: after 1 and 3 days of alkaline stress, RL decreased by approximately 57% and 73%, respectively. Additionally, the coefficient of variation (CV) under treatment was notably higher than that under control, indicating substantial genotypic differences in alkaline stress responses (Table 1).

**Figure 1:**
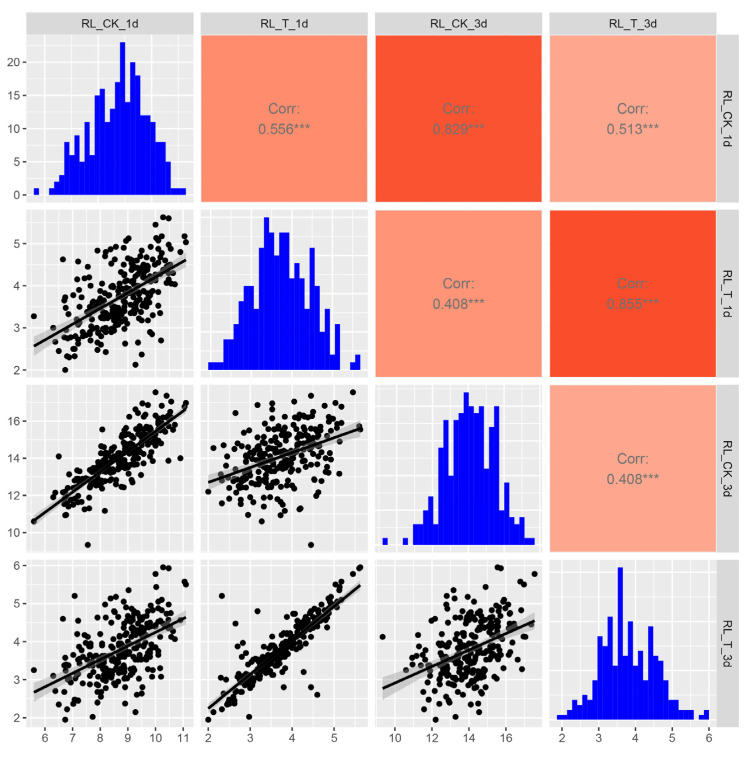
Scatter plot matrix of alkaline stress phenotypes in 258 wheat accessions. Note: RL, root length; CK, control; T, treatment. The asterisks (***) indicate a statistically significant difference between groups (*p* < 0.001).

Correlation analysis revealed strong associations between the same trait across different time points and treatments; for example, significant positive correlations were observed between root length measurements at different time points, with r = 0.829 under control conditions (RL_CK_1d vs. RL_CK_3d) and r = 0.855 for treated samples (RL_T_1d vs. RL_T_3d) (Figure 1). These findings suggest that RL maintains stable genetic performance over time. Under alkaline stress, the correlation between RL_T_1d and RL_CK_1d decreased significantly (r = 0.408), with a further 1.5-fold reduction observed after 3 days of treatment (Figure 1). These results demonstrate that RL is highly sensitive to alkaline stress, making it a key phenotypic indicator for evaluating alkaline stress responses in wheat.

### 2.2. Population Structure Analysis

**Figure 2:**
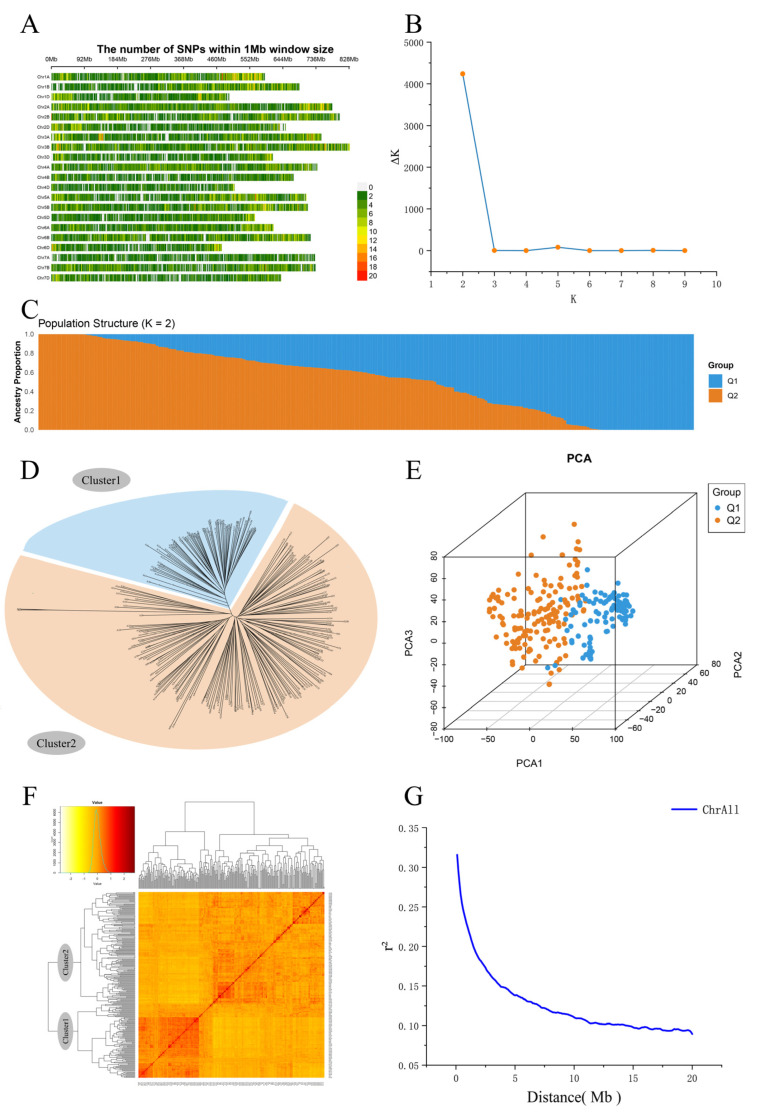
Genetic diversity and population structure analysis on 258 wheat accessions. (**A**) SNP density; (**B**) estimated delta K in structure analysis; (**C**) two subgroups inferred from structure analysis; (**D**) a Neighbor-Joining (NJ) phylogenetic tree; (**E**) principal component analysis (PCA); (**F**) kinship matrix-based cluster analysis; (**G**) linkage disequilibrium (LD) decay.

This study conducted genetic diversity and population structure analysis on 258 wheat accessions based on 36,044 genome-wide polymorphic SNP markers. First, the distribution of SNP density was calculated for each 1 Mb genomic interval, revealing a relatively balanced distribution of markers across chromosomes (Figure 2A).

Population structure analysis was performed using the Structure software (v2.3.4). The ΔK value reached its peak at K = 2, indicating that the natural population could be divided into two major subpopulations (Q1 and Q2) (Figure 2B). The structure bar plot clearly displayed the differentiation pattern between Q1 and Q2, with some accessions showing mixed genetic components, suggesting possible historical gene flow or hybridization events (Figure 2C).

To further validate the robustness of the subpopulation classification, a Neighbor-Joining (NJ) phylogenetic tree was constructed; the resulting tree showed high consistency with the structure analysis, indicating clear clustering between the two subpopulations (Figure 2D). Principal component analysis (PCA) further confirmed the genetic differentiation among accessions along the PC1 and PC2 axes (Figure 2E), aligning with both the results of the population structure and the phylogenetic tree.

Kinship matrix analysis (Figure 2F) indicated that most accessions exhibited weak genetic relatedness, with only a few varieties showing higher kinship. To assess the linkage disequilibrium (LD) decay level in the population, the relationship between the r^2^ value and physical distance was plotted across the genome. The results showed that when r^2^ dropped to 0.2, the LD decay distance was approximately 1.5 Mb (Figure 2G), suggesting rapid LD decay in this natural population and providing a reliable foundation for subsequent functional gene discovery and validation.

### 2.3. GWAS Candidate Gene Prediction

**Figure 3:**
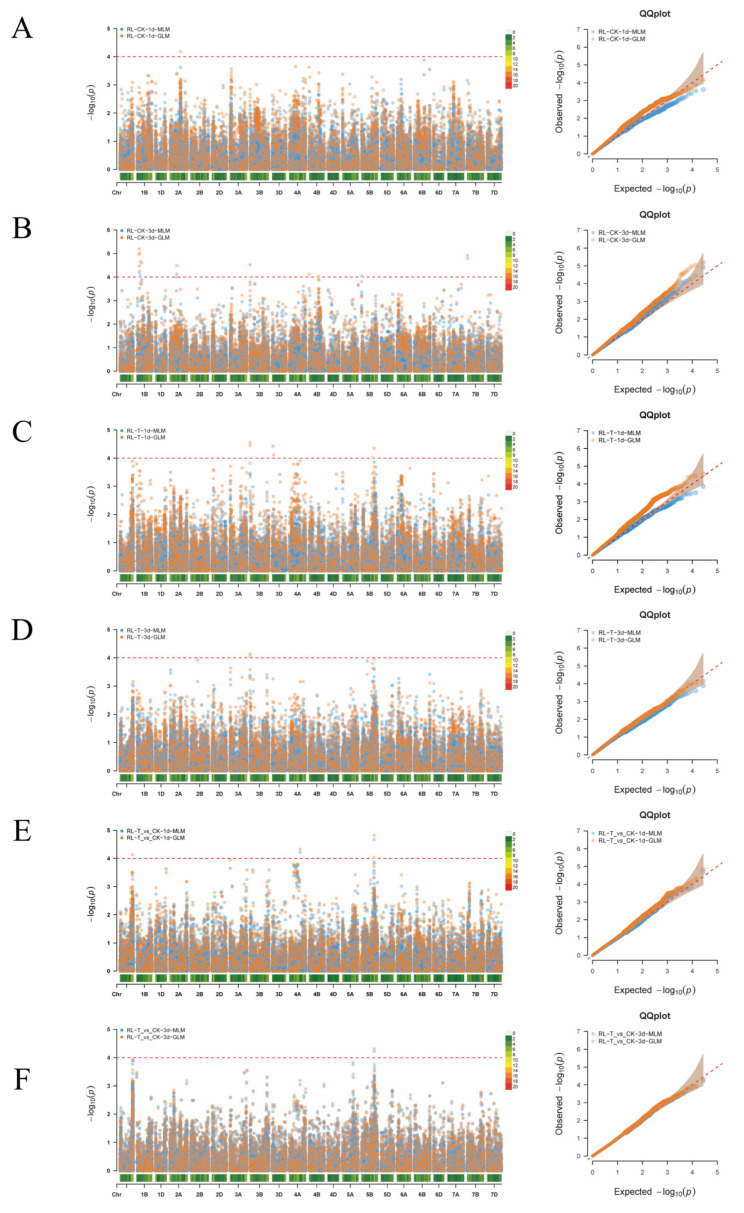
Manhattan (**left**) and Q–Q (**right**) plots for alkaline stress-related traits in 258 wheat accessions using the mixed linear model (MLM) and the general linear model (GLM). (**A**) Root length at 1 day under control conditions; (**B**) root length at 3 days under control conditions; (**C**) root length at 1 day under alkaline stress; (**D**) root length at 3 days under alkaline stress; (**E**) relative root length at 1 day; (**F**) relative root length at 3 days. Horizontal lines indicate genome-wide significance thresholds.

In this study, a genome-wide association study (GWAS) was performed on 258 natural wheat accessions using 36,044 SNP markers to analyze root length (RL) traits under control (CK), alkaline stress (T), and stress/control ratio (T/CK) conditions at 1 and 3 days after-treatment. Two complementary statistical approaches—the general linear model (GLM) and mixed linear model (MLM)—were implemented for association analysis, with results visualized through Manhattan and quantile–quantile (Q–Q) plots (Figure 3). As shown in Figure 3A, the Manhattan plot identified 22 loci significantly associated with root length at the genome-wide level. Among these, the peak SNP on Chr2A (2A_313405793) reached extreme significance, with its −log_10_(P) value exceeding 4. The sample group RL-CK-3d contained the highest number of loci with a total of 12, followed by the 1-day alkaline stress treatment group with 6 loci.

The Q–Q plot (Figure 3B) demonstrates that the observed distribution of P-values largely aligns with the expected uniform distribution, suggesting effective control of the population structure. In the tail region, a modest upward deviation in the observed values from the expected line indicates the presence of true genetic signals rather than false positives. The absence of widespread outliers confirms sufficient model calibration.

GWAS analysis enabled the identification of nine significant QTLs, including six for absolute root length under alkaline stress and three for relative root length, distributed across chromosomes 1A, 3B, 3D, 4A, and 5B (Table 2).

We selected two representative SNP loci for phenotypic comparison, including 2A_313405793 (identified under control conditions) and 3B_704997 (under alkaline stress). Both loci were significantly associated with root length and exhibited relatively high R^2^ values, supporting their relevance in explaining the phenotypic variation among genotypes. The boxplots (Supplementary Figure S1) clearly show statistically significant differences in root length among the homozygous and heterozygous genotypes (e.g., GG vs. CC, TT vs. CC), supporting the association between allelic variation and phenotypic expression at the juvenile stage.

At 1 d after treatment, six significant loci were detected on 3B (2), 3D (1), and 5B (3) (Table 2). At 3 d after treatment, three significant loci were identified on 3B (2) and 5B (1), all of which overlapped with those detected at 1 d (Table 2). Within the 3 Mb genomic regions flanking these nine loci we identified 285 candidate genes, including: (i) 39 transcription factors (TFs) comprising Zinc finger (8), bHLH (9), and Homeobox (6) families; (ii) 7 transporter/ion channel genes; and (iii) 15 hormone-related genes (Supplementary Table S2). These candidate genes may play crucial roles in the regulation of root growth and stress responses, providing potential targets for further functional validation.

### 2.4. Phenotypic Analysis of Two Genotypes with Significant Differences in Alkaline Tolerance

**Figure 4:**
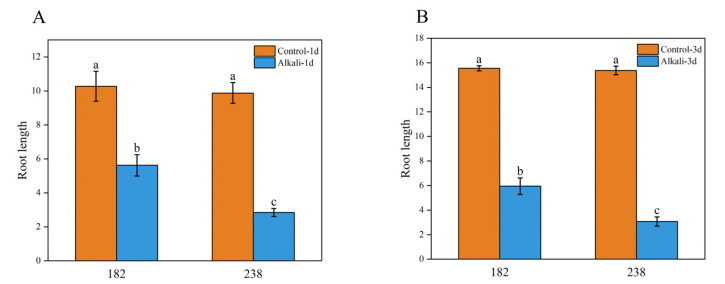
Phenotypes of wheat seedlings under control and alkaline stress conditions. (**A**) Treatment with alkaline stress for one day; (**B**) treatment with alkaline stress for three days. Different letters above bars indicate significant differences at *p* < 0.05, according to Duncan’s multiple range test.

**Figure 5:**
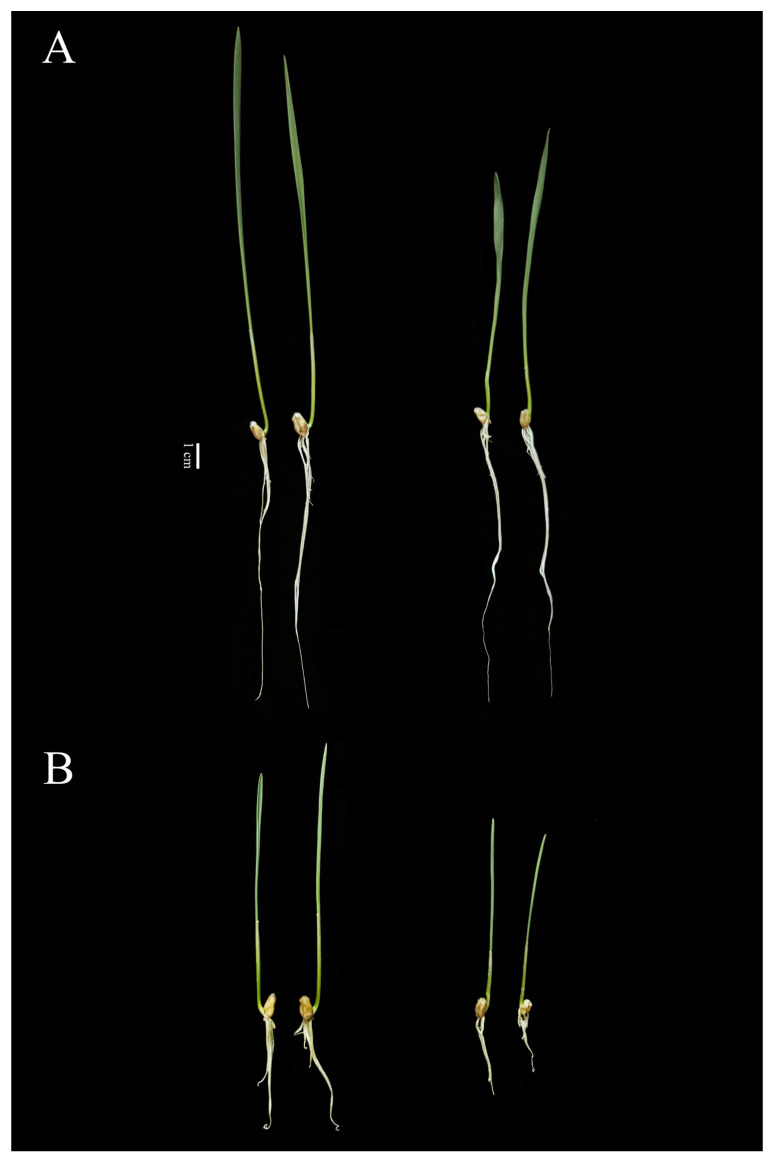
Morphological comparison of two wheat genotypes under control and alkaline stress conditions. Seedlings of genotype 182 (**left**) and genotype 238 (**right**) were photographed on the third day after treatment. (**A**) Control group (CK); (**B**) alkaline stress group. Alkaline stress was induced using a mixed carbonate solution of NaHCO_3_ and Na_2_CO_3_ at a 9:1 molar ratio. Bar = 1 cm.

Under alkaline stress conditions, genotypes 182 and 238 exhibited significant growth differences (Figure 4 and Figure 5). In the control group, genotype 182 showed significantly longer roots than that in the stress-treated group (Figure 4A). After alkaline stress treatment at 1 d, its root length decreased by approximately 45% compared to the control, indicating moderate growth inhibition. In contrast, genotype 238 was more severely affected by alkaline stress, with a 71% reduction in root length, demonstrating pronounced growth suppression (Figure 4B). A similar trend was observed on the third day of stress treatment, with the degree of inhibition in genotype 238 being significantly higher than that in genotype 182. Given their distinct phenotypic responses, 182 represents a typical alkaline-tolerant genotype, while 238 is a typical alkaline-sensitive genotype (Figure 5). Therefore, these two wheat materials were selected as representative genotypes for subsequent transcriptome sequencing analysis.

**Figure 6:**
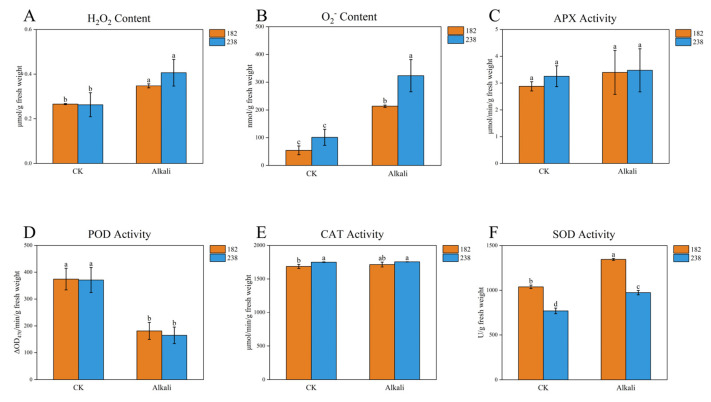
Reactive oxygen species (ROS) contents and antioxidant enzyme activities. in roots of genotypes 182 (alkaline-tolerant) and 238 (alkaline-sensitive) seedlings under control (CK) and alkaline stress conditions. (**A**) Hydrogen peroxide (H_2_O_2_) content; (**B**) superoxide anion (O_2_^−^) content; (**C**) ascorbate peroxidase (APX) activity; (**D**) peroxidase (POD) activity; (**E**) catalase (CAT) activity; (**F**) superoxide dismutase (SOD) activity. Roots were sampled from seedlings on the third day after treatment with a mixed carbonate solution composed of NaHCO_3_ and Na_2_CO_3_ at a 9:1 molar ratio. Data are presented as means ± SD (*n* = 3). Different letters above bars indicate significant differences at *p* < 0.05, according to Duncan’s multiple range test.

To determine whether there were differences in antioxidant capacity between the two genotypes, the levels of H_2_O_2_ and O_2_^−^ were measured. As shown in Figure 6, no significant differences were observed under control conditions. Under alkaline stress, both indicators increased, with significantly higher accumulation in genotype 238 than in 182. Additionally, we examined the activities of several antioxidant enzymes, among which APX and CAT showed no significant differences between treated and control samples (Figure 6). Under alkaline stress, POD activity decreased, although no significant difference was detected between the two genotypes. Notably, SOD activity significantly increased under alkaline stress compared to the control, with a more pronounced increase in genotype 182 than in 238 (Figure 6). In conclusion, we infer that genotype 182 exhibits stronger antioxidant capacity than genotype 238 under alkaline stress.

**Figure 7:**
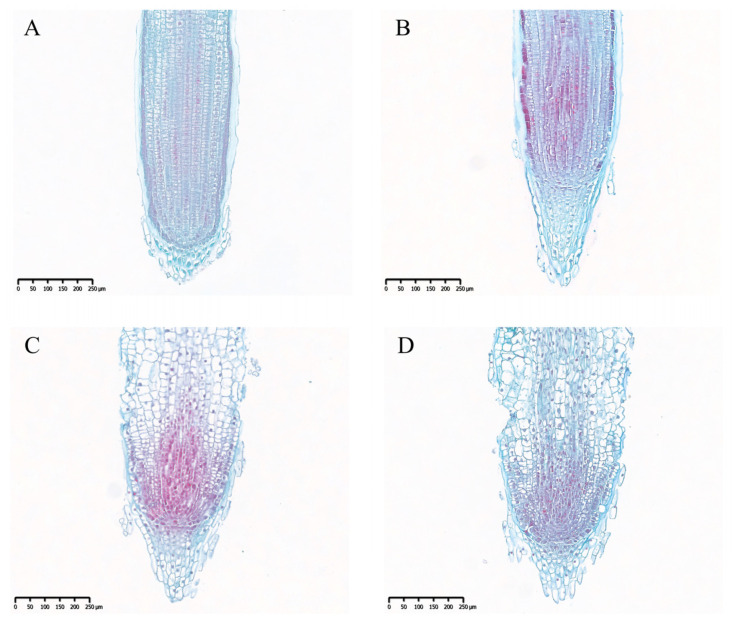
Microphotographs are presented showing the root tip architecture of two wheat genotypes under control and alkaline stress treatments after 3 days. Control treatment (CK-3d): genotype 182 (**A**), genotype 238 (**B**); alkaline stress treatment (T-3d): genotype 182 (**C**), genotype 238 (**D**). Paraffin sections of root tips were stained with safranin-fast green. Scale bars = 250 μm.

As shown in Figure 7, compared to genotype 238, the cells in the root cap zone and meristematic zone of genotype 182 were arranged more regularly and compactly, with fewer damaged cells and better tissue integrity. In genotype 238, most cells in the elongation zone were damaged, exhibiting irregular morphology and a loosely disordered arrangement, with the epidermal cells being completely absent (Figure 7). These observations suggest that genotype 238 is more affected by alkaline stress than genotype 182.

### 2.5. RNA-Seq Analysis Results

**Figure 8:**
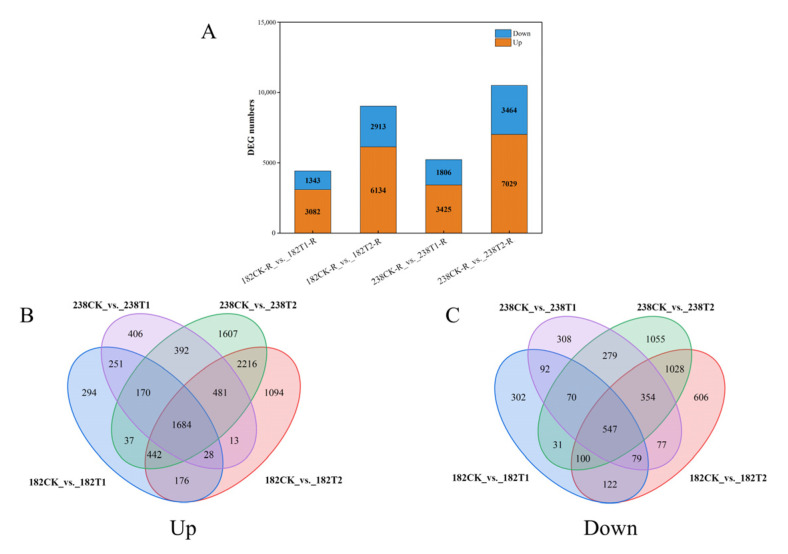
The number of differentially expressed genes (DEGs) between two genotypes after 1 and 3 days of alkaline stress. (**A**) The number of DEGs; (**B**) up-regulated genes; (**C**) down-regulated genes.

The results showed that the number of differentially expressed genes (DEGs) in both genotypes exhibited significant increases with prolonged stress treatment duration (Figure 8A). Compared to 1 day of treatment, the number of DEGs after 3 days of treatment increased by 4622 and 5262 in genotypes 182 and 238, respectively (Figure 8A). Overall, regardless of whether the treatment lasted 1 or 3 days, the number of up-regulated genes was higher than that of down-regulated genes. Between the two genotypes, genotype 182 had fewer up- and down-regulated genes at both time points (Figure 8A).

A total of 1684 genes were consistently up-regulated under alkaline stress in both genotypes at both time points (Figure 8B, Supplementary Table S3). These genes were primarily involved in key biological processes such as ion homeostasis regulation, oxidative stress defense, transcriptional reprogramming, and cell wall modification. Among the transporter-encoding genes, Potassium transporter 24 (*TraesCS7B03G0303400*) and ABC transporter C family members (e.g., *TraesCS7A03G0281300*, *TraesCS3D03G0274200*, *TraesCS7D03G0255700*) may cooperatively regulate ion homeostasis, while Patatin-like protein 2 (*TraesCS4D03G0676000*) and Lipase-like PAD4 (*TraesCS4B01G100100*) might enhance alkaline tolerance by maintaining membrane lipid stability. In the oxidative defense system, the significant up-regulation of genes such as peroxidase (*TraesCS7B03G0665000*) and glutathione S-transferase (*TraesCS6A03G0329800*) indicated the establishment of an efficient reactive oxygen species (ROS) scavenging mechanism. In terms of transcriptional regulation, identified transcription factors included WRKY (*TraesCS2A03G0334500*) and bHLH (*TraesCS5D03G0872500*), which may enhance adaptability through a regulatory cascade affecting downstream stress-responsive genes. Additionally, changes in the expression of cell wall modification-related genes such as expansin (*TraesCS1A03G0559000*, *TraesCS3A03G0771000*) and chitinase (*TraesCS2D03G0798500*) suggest that dynamic remodeling of cell wall structure could be an important strategy for coping with alkaline stress. Additionally, several genes involved in pH regulation were identified, such as proton ATPase (*TraesCS2D03G1099700*) and organic acid secretion-related genes (*TraesCS6A03G0613600*, *TraesCS2B03G1015200*). Notably, approximately 11% of the up-regulated genes potentially harboring novel alkaline tolerance-related regulatory mechanisms, such as *NewGene_30859*, remain functionally uncharacterized.

A total of 547 common down-regulated genes under alkaline stress were identified, meaning that these genes were down-regulated in both genotypes at both treatment time points (Figure 8C, Supplementary Table S4). For example, photosynthesis-related genes (including components of photosystem I/II, chlorophyll synthases, and electron transport chain proteins) reflected the significant suppression of energy capture processes under alkaline stress. Genes such as *TraesCS5D03G0511400* (aldehyde dehydrogenase) and *TraesCS7D03G1052300* (carbonate dehydratase) showed reduced expression, directly impairing the efficient conversion of light energy. The down-regulation of cell growth and division-related genes (including cyclins, tubulins, and proliferation-associated kinases) suggested a marked reduction in cell division activity. Specific genes such as *TraesCS7D03G0740200* (DNA polymerase α) were also down-regulated, hindering cell cycle progression. Down-regulation of nutrient transport-related genes (including amino acid permeases, sugar transporters, and ion channels) indicated possible adjustments in resource allocation under alkaline stress. Genes such as *TraesCS6A03G0422000* (encoding a transmembrane amino acid transporter protein) and *TraesCS6A03G0074400* (encoding a high-affinity nitrate transporter) showed reduced expression, potentially limiting nitrogen utilization and carbon allocation. Stress-responsive genes—such as those associated with pathogenesis-related proteins (*TraesCS5A03G0229500*) and heat-shock proteins—were also down-regulated, suggesting the suppression of certain biotic stress-response pathways. Notably, 71 genes (approximately 13%)—such as *NewGene_30840* and *NewGene_4168*—remain uncharacterized, implying the existence of unknown alkaline-tolerance regulatory mechanisms.

### 2.6. Common Genes Identified via GWAS and RNA-Seq

Integrating the results of the GWAS and RNA-seq analyses, seven common genes were identified, six of which were up-regulated and one down-regulated (Table 3). Among them, three genes encode transcription factors: *TraesCS1A03G0917700* (Myb-related protein 38), *TraesCS3D03G0225500* (Zinc finger CCCH protein 2), and *TraesCS5B03G0961700* (Transcription factor bHLH148) (Table 3). These genes may enhance resistance by regulating the expression of stress-related genes. *TraesCS3D03G0226000* (ATP-dependent 6-phosphofructokinase 3, ATP-PFK 3) and *TraesCS5B03G0953100* (Organic cation/carnitine transporter 7, OCT7) have been hypothesized to be involved in energy metabolism reprogramming and osmotic substance transport to maintain cellular homeostasis, respectively. Additionally, *TraesCS1A03G0913800* (Beta-1,3-galactosyltransferase pvg3) and *TraesCS3D03G0227400* (Profilin) are associated with cell wall modification and cytoskeleton stabilization, thereby directly defending against mechanical or osmotic damage.

### 2.7. Quantitative Real-Time PCR (qPCR)

**Figure 9:**
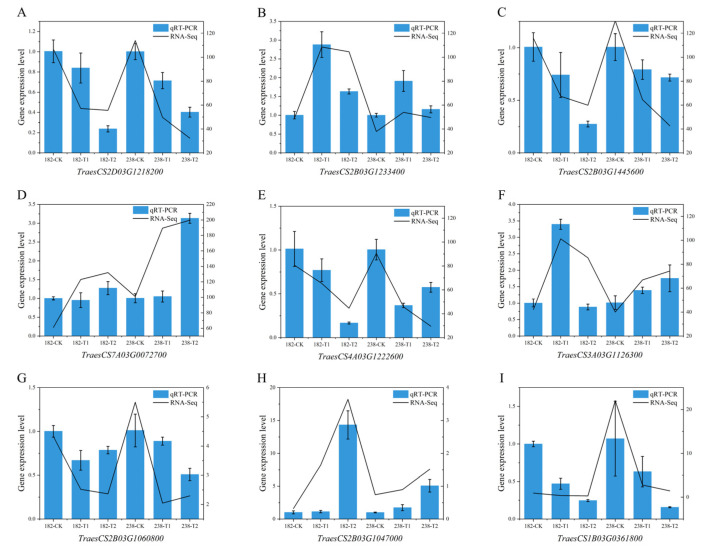
Quantitative real-time PCR (qRT-PCR) validation of transcript levels for nine selected genes based on RNA-seq analysis. (**A**) TraesCS2D03G1218200, (**B**) TraesCS2B03G1233400, (**C**) TraesCS2B03G1445600, (**D**) TraesCS7A03G0072700, (**E**) TraesCS4A03G1222600, (**F**) TraesCS3A03G1126300, (**G**) TraesCS2B03G1060800, (**H**) TraesCS2B03G1047000, (**I**) TraesCS1B03G0361800. Error bars represent the standard deviation (*n* = 3).

Nine differentially expressed genes were randomly selected for qRT-PCR analysis. The results showed that the expression of the *TraesCS2B03G1233400* gene was induced and up-regulated under alkaline stress. Its fold increase in genotype 182 was higher than that in 238, being 2-fold and 1.5-fold, respectively (Figure 9). A similar expression pattern was also observed for *TraesCS3A03G1126300* and *TraesCS2B03G1047000*. In contrast, the fold increase in *TraesCS7A03G0072700* in genotype 238 was approximately twice that in 182. Furthermore, five genes (*TraesCS2D03G1218200, TraesCS2B03G1445600, TraesCS4A03G1222600, TraesCS2B03G1060800*, and *TraesCS1B03G0361800*) were all down-regulated under alkaline stress treatment (Figure 9). Overall, the gene expression changes revealed by the qRT-PCR results were strongly consistent with the RNA-seq data, confirming the reliability of the sequencing results.

## 3. Discussion

### 3.1. Root Growth Is One of the Important Indicators for Evaluating Alkaline Tolerance

The root system is a crucial organ for perceiving changes in the soil environment, particularly under conditions such as salt, drought, and alkaline stress [21]. Therefore, studying how roots recognize environmental signals and the molecular basis of root changes under stress is of great significance for understanding the adaptability of plants to environmental conditions. Research has shown that alkaline stress inhibits root growth more significant than salt stress; for instance, after 15 days of alkaline stress treatment, the root growth of two apple rootstock varieties was significantly suppressed [22]. Compared to control seedlings, their total root length, surface area, and volume decreased, although the extent of reduction varied between alkaline-tolerant and -sensitive varieties [22]. Similarly, Liu et al. evaluated the alkaline tolerance of eight soybean genotypes with varying root systems and found that most root traits exhibited an overall declining trend, except for root diameter and the biomass allocation ratio (shoot/root) [23]. In rice, alkaline stress has been observed to affect the fresh weight, length, and number of roots [24]. In this study, we also found that the primary root length of wheat was inhibited to varying degrees under alkaline stress, with significant genotypic differences (Figure 1). Meanwhile, it was found that the root inhibition effect in alkaline-tolerant genotypes was significantly greater than that in sensitive ones when comparing their root phenotypes (Figure 4 and Figure 5). Furthermore, at the cellular level, it was observed that the root systems of sensitive genotypes exhibited markedly poorer cellular integrity when compared to those of tolerant genotypes (Figure 7). These findings are consistent with other studies reporting on alkaline stress [25]. Evidently, the growth status of the root system serves as an important indicator for assessing alkaline tolerance. We identified several superior genotypes which are capable of maintaining relatively high root length, laying a foundation for further research into the mechanisms of alkaline tolerance.

### 3.2. The Antioxidant Capacity Is a Key Indicator for Assessing Alkaline Stress Tolerance

Saline–alkaline stress induces osmotic and ionic stress, which in turn promotes the overproduction and accumulation of reactive oxygen species (ROS), including hydrogen peroxide (H_2_O_2_), superoxide anion (O_2_^−^), and hydroxyl radicals (OH.) [10,24]. In response, plants initiate various adaptive mechanisms, such as enhancing the activities of antioxidant enzymes—including superoxide dismutase (SOD), catalase (CAT), ascorbate peroxidase (APX), and peroxidase (POD)—along with the activation of non-enzymatic antioxidant systems. Together, these elements function to neutralize ROS, reduce oxidative harm, and maintain the stability of cellular membranes [10,24]. Alkaline treatment has been shown to induce a substantial accumulation of ROS, including O^2–^ and H_2_O_2_, in rice [24]. A key finding was that the tolerant cultivar exhibited significantly less ROS accumulation than the sensitive cultivar [24]. Under alkaline stress, both O_2_^−^ and H_2_O_2_ levels were increased in two wheat cultivars [25]: while they accumulated similar amounts of ROS under control treatment, SR4 exhibited lower ROS accumulation than JN177 when exposed to alkaline conditions [25]. In this study, we similarly observed increases in the levels of both aforementioned ROS under alkaline stress, with the tolerant genotype 182 exhibiting lower accumulation than the sensitive genotype 238 (Figure 6). Under alkaline conditions, the root tissues exhibited marked increases in the activities of key antioxidant enzymes, including SOD, POD, CAT, and APX. It is worth noting, however, that the increase in APX activity in the tolerant genotype was not statistically significant. Regarding the ROS scavenging enzymes SOD, APX, CAT, and POD, CAT previously exhibited consistently greater activity in SR4 compared to JN177 [25]. Under stress conditions, the activities of SOD and APX were suppressed; however, SR4 maintained higher SOD and APX activity levels than JN177. In contrast, POD activity remained unchanged by stress in SR4 but was elevated in JN177 [25]. In our study, no significant differences in APX and CAT activities were observed between the treated and control groups. While a reduction in POD activity was observed under alkaline stress, the difference between the two genotypes was not significant. In contrast, SOD activity exhibited a marked increase under stress conditions relative to the control, with genotype 182 showing a more substantial rise than genotype 238 (Figure 6). Therefore, the disparity in antioxidant capacity under alkaline conditions is a key metric for evaluating stress tolerance.

### 3.3. The Major Loci Associated with Alkaline Stress Tolerance Identified in Wheat

GWAS and QTL mapping have become pivotal strategies for deciphering the genetic mechanisms of salt tolerance in crops, particularly in the genetic regulation of root morphology traits (e.g., root length, fresh weight, and dry weight) [26,27]. As a salt-sensitive crop, salt tolerance-related loci have been extensively identified across various genetic populations of wheat. In terms of GWAS research, a GWAS analysis of 125 wheat landraces and cultivars identified three significant loci on chromosomes 6B, 7A, and 7D that regulate RL, RFW, and RDW under salt stress, respectively [28]. Further studies have shown that root-related loci are predominantly enriched in the A and B genomes, with RFW-associated loci concentrated on 4A, 5A, and 5B; RDW loci distributed on 5A and 5B; and RL regulatory loci mapped to chromosome 7A [29]. Regarding QTL mapping, based on an RIL population derived from ZM175 × XY60, five QTLs for maximum root length (MRL) under salt stress have been identified, which are located on chromosomes 2A, 2B, 2D, 3B, and 4A [30]. In an RIL population from Xiaoyan 54 × Jing 411, two main root length (MRL) QTLs were detected under salt stress—qMRL.ST-4D and qMRL.ST-6B—explaining 8.3% and 16% phenotypic variation, respectively [31]. In contrast, genetic research on alkaline stress tolerance remains significantly underdeveloped. Although GWAS or QTL studies have been reported in rice [32], maize [33], and rapeseed [34], the molecular mechanisms of alkaline tolerance in wheat still lack systematic investigation. In this study, we identified nine loci distributed across five chromosomes associated with alkaline tolerance (Table 2). Among them, the identified loci on 3D and 5B overlap with previously reported QTLs associated with the salt stress response. Meanwhile, several novel loci which are responsive to alkaline stress were also identified; for example, the locus located on chromosome 3B controls root length under alkaline stress and accounts for up to 6.54% of the explained phenotypic variation (Table 2). This breakthrough not only fills a critical knowledge gap in wheat alkaline stress genetics, but also provides valuable targets for subsequent cloning of alkaline tolerance genes and mechanistic studies. It is generally considered that loci explaining ≥10% of phenotypic variation are classified as large-effect loci, whereas most of the alkaline stress-responsive loci identified in this study fall within the range of 3–10%, representing moderate-effect loci. These loci are likely involved in polygenic regulatory networks and require synergistic interactions with other loci to exert their effects. Therefore, in breeding strategies, they can be utilized in gene pyramiding—that is, stacking multiple effect loci via hybridization or molecular design.

### 3.4. Promising Candidate Genes Conferring Alkaline Tolerance Were Identified in Wheat

The impacts of alkaline stress on plants span multiple levels. Initially, it alters the osmotic pressure within plant cells, thereby triggering osmotic stress. Concurrently, the persistent accumulation of reactive oxygen species under alkaline stress gives rise to oxidative stress. Moreover, the detrimental effects of alkaline stress on plants are further manifested in the disruptions caused by high pH levels and the perturbation of ion homeostasis. In response, plants have evolved a range of adaptive strategies. Thus, at the molecular level, genes involved in plant responses to alkaline stress primarily fall into the following categories: Osmotic regulation [35,36], pH regulation [37,38], antioxidant defense [38], and ion regulation [39]. For instance, the wheat gene *TaALMT1* is hypothesized to contribute to alkaline tolerance by mediating the efflux of malic acid and GABA—processes that may be coupled with proton extrusion [40]. Additionally, alterations in genes associated with signal perception and transduction during alkaline stress responses have been increasingly identified [14]. Among these, transcription factor genes play pivotal roles in adaptation to alkaline stress, with key families including WRKY, MYB, AP2/EREBP, bZIP, and NAC. In this study, a total of 285 candidate genes were predicted Via GWAS analysis; furthermore, 2231 differentially expressed genes (DEGs) were found to be induced by alkaline stress in both cultivars at both treatment time points (Figure 5, Supplementary Table S2). Among them, 1684 were up-regulated and 547 were down-regulated, respectively. Integrating the GWAS and RNA-seq results, seven co-expressed genes were identified in total (Table 3). Among these, a gene encoding an MYB transcription factor (*TraesCS1A03G0917700*) exhibits homology to the rice *OsMYB55* gene [41], which belongs to the R2R3-MYBs subfamily and is closely associated with plant stress resistance [42]. When this gene was expressed in maize, the plant’s tolerance to high temperature and drought was significantly enhanced, primarily through the induction of stress-responsive gene expression [43]. Among these, antioxidant-related genes such as phosphoadenosine phosphosulfate (PAPS) reductase and Ferredoxin-3 were markedly up-regulated [41]. Furthermore, the expression level of *TraesCS1A03G0917700* under alkaline stress was found to vary across different varieties, with higher levels observed in tolerant varieties compared to sensitive ones. Based on these findings, we can speculate that *TraesCS1A03G0917700* may enhance alkaline tolerance by regulating downstream networks (e.g., ROS scavenging pathways); in this regard, functional verification will be conducted in subsequent studies.

**Figure 10:**
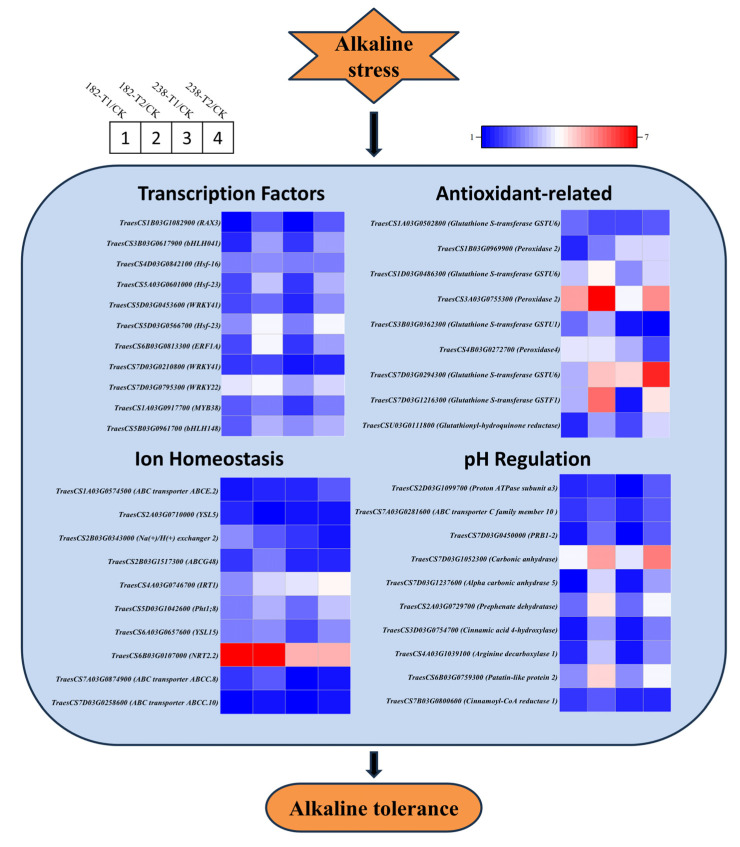
Elucidating the molecular hypothesis underlying wheat’s response to alkaline stress.

Based on our findings, we propose a molecular model for the alkaline stress response in wheat (Figure 10). During alkaline stress, transcription factors act as key intermediaries that connect sensory signals with genetic responses. They react to upstream stimuli and directly influence the expression of stress-related genes through binding to specific cis-acting elements. Numerous transcription factors from prominent families—including bHLH, AP2/ERF, NAC, MYB, and WRKY—were found to exhibit marked changes in expression under alkaline conditions (Figure 10). Many members of these families have been implicated in plant responses to abiotic stresses. The alkaline stress response mechanism primarily encompasses the following aspects: enhancing resistance to oxidative damage by regulating antioxidant-related genes such as glutathione S-transferase and peroxidase; maintaining ion homeostasis through channel proteins and transporters such as high-affinity nitrate transporters, ammonium transporters, calcium-transporting ATPases, and cation/H^+^ antiporters; and preserving intracellular pH stability via the secretion and synthesis of organic acids along with other regulators of hydrogen ion concentration, including V-type proton ATPase subunits and carbonic anhydrase (Figure 10).

In conclusion, the root system—as a key organ for perceiving soil environmental changes—plays a critical role in the adaptation of plants to stresses such as alkaline stress. This study confirmed that alkaline stress significantly inhibits primary root growth in wheat with distinct genotypic differences, and identified superior germplasms that maintain relatively higher root length, providing valuable germplasm resources for the exploration of alkaline tolerance mechanisms. We identified alkaline tolerance-related genetic loci in wheat, as well as candidate genes associated with alkaline stress responses, including those related to osmotic regulation, pH balance, antioxidant defense, and ion homeostasis. We identified co-expressed genes by integrating the GWAS and RNA-seq results, indicating the potential roles of these genes in enhancing alkaline tolerance. Overall, this study provides insights into the physiological, genetic, and molecular aspects of wheat root responses to alkaline stress, laying a foundation for improving alkaline tolerance in wheat through targeted germplasm innovation and molecular breeding.

## 4. Materials and Methods

### 4.1. Experimental Materials

This study employed 258 natural wheat accessions as experimental materials for integrated GWAS and RNA-seq analyses. The panel included representative cultivars and local landraces from China’s major wheat-producing regions, along with internationally sourced wheat germplasms (Supplementary Table S1). For transcriptome sequencing (RNA-seq) analysis, two contrasting genotypes were selected based on their differential responses to alkaline stress in preliminary phenotypic evaluations: the alkaline-tolerant cultivar Dingxi 38 (accession 182) and the alkaline-sensitive line TDP.D-27 (accession 238). These well-characterized materials were established as reference genotypes for comparative transcriptomic studies, enabling the systematic identification of differentially expressed genes (DEGs) and subsequent discovery of alkaline stress-responsive candidate genes.

### 4.2. Hydroponic Experiment

This study carried out alkaline stress treatment and phenotypic evaluation of wheat seedlings using a controlled hydroponic system. The seeds were first soaked in 3% hydrogen peroxide (H_2_O_2_) for 15 min and then thoroughly rinsed with sterile distilled water. The seeds were placed in moist Petri dishes and germinated in an artificial climate chamber in the dark at 25 °C. When the radicle length reached approximately 1 cm, the seedlings were transferred to 96-well hydroponic boxes for cultivation at 25/18 °C (day/night) with 70% relative humidity and a photoperiod of 12 h. Each genotype was set up with four biological replicates, and the nutrient solution was replaced daily. Alkaline stress treatment began on the second day after transplantation, using a mixed carbonate solution of NaHCO_3_ and Na_2_CO_3_ at a molar ratio of 9:1 [41].

### 4.3. Phenotypic Measurement

To evaluate the phenotypic responses of different wheat materials under alkaline stress, seedlings were sampled on the first and third days after treatment. Key growth traits were measured at these times, including root length (RL). Each genotype had four biological replicates, and the average value of independent measurements was used as the phenotypic indicator for variation analysis and the subsequent genome-wide association study (GWAS).

The determination of hydrogen peroxide (H_2_O_2_) content was performed according to the operating instructions for the kit provided by Jiangsu Aidisheng Biological Technology Co., Ltd. (Nanjing, China). CAT, SOD, and POD activities were measured using kits provided by Solarbio (Solarbio, Beijing, China). The O_2_^−^ content was assayed by Nanjing Convinced-test Technology Co., Ltd. (Nanjing, China). The determination of these physiological parameters can also be performed according to the method described by Meng et al. (2017) [25].

The hydroponic cultivation and stress treatment methods remained consistent with the previous protocol. After 3 days of alkaline stress treatment, the main roots of both control and alkaline-stressed samples were fixed in FAA solution for 24 h, followed by immersion in 50% ethanol for an additional 24 h. A 0.5 cm apical segment of the main root was excised, dehydrated through a graded ethanol series, and cleared using tertiary butanol and xylene. The root tips were infiltrated with wax and embedded using an embedding machine, and longitudinal sections were prepared on glass slides. Staining, sectioning, and microscopic observation procedures were performed according to the methods described by Thangthong et al. (2019) [44].

### 4.4. GWAS Analysis

After organizing the phenotypic data, GWAS analysis was performed using the TASSEL v5.0 software platform. In addition to the general linear model (GLM), a mixed linear model (MLM) was applied, incorporating population structure (Q) and kinship matrix (K) for correction to reduce interference due to false positives [45,46]. The significance threshold for multiple comparisons was adjusted using the Bonferroni–Holm correction method. Based on the SNP marker quality control standards and population sample size in this study, the significance screening threshold was set at −log_10_(P) ≥ 4.0, corresponding to P ≤ 1.0 × 10^−4^. This threshold was used to identify significant SNP loci associated with alkaline stress. The association results were visualized using the R (v4.4.2) package CMplot (v4.5.1) to generate Manhattan plots and Q–Q plots, displaying significant association signals and data fitting.

The chromosomal and physical positions of significantly associated SNP loci were mapped using the wheat reference genome (IWGSC RefSeq v1.1), and candidate genes within a 1.5 Mb upstream and downstream interval were retrieved. Potential functional genes related to alkaline stress were further screened based on gene annotation information and functional databases.

### 4.5. RNA-Seq Analysis

The hydroponic cultivation and alkaline stress treatment methods were identical to those previously described, with an unstressed group serving as the control. A total of 18 root RNA-seq samples were obtained, including two genotypes (182 and 238), three treatment time points (0 h, 6 h, and 24 h), and three biological replicates. All root samples were immediately frozen in liquid nitrogen after treatment and stored at −80 °C until subsequent RNA extraction.

Library construction was performed by Beijing Biomarker Technologies Co., Ltd. (Beijing, China). Prior to library preparation, total RNA was evaluated for purity, concentration, and integrity. mRNA was enriched using Oligo(dT)-coupled magnetic beads and subsequently fragmented randomly. First-strand cDNA synthesis was performed, followed by second-strand synthesis to generate double-stranded cDNA. The resulting cDNA fragments were subjected to end repair, 3′-adenylation, adapter ligation, and PCR amplification to construct the final sequencing libraries. Library quality control included fragment size distribution analysis using the Qsep400 system, while quantification was performed using Qubit 3.0 and qPCR.

Qualified libraries were sequenced on the Illumina NovaSeq 6000 platform (PE150 mode), with each sample yielding at least 5.7 Gb of high-quality data. Raw data were quality-controlled using Fastp v0.23.4 to filter out adapter-contaminated sequences, low-quality sequences (Q ≤ 10 for over 50% of bases), and reads with >10% N bases, resulting in clean reads.

Clean reads were aligned to the wheat reference genome (*Triticum aestivum*, IWGSC RefSeq v2.1) using HISAT2 v2.2.1 [47]. The reads were then assembled using StringTie (v2.2.1), and gene structures were optimized via novel transcript prediction based on the alignment results. Expression levels were normalized as FPKM (Fragments Per Kilobase of transcript per Million mapped reads), and read counts were quantified using featureCounts (v2.0.3). Differential expression analysis was performed using DESeq2 v1.46.0, with the screening criteria set at |log_2_FC| ≥ 1, FDR < 0.01, and FPKM ≥ 1. Based on inter-group differences in expression, lists of significantly up- and down-regulated genes were obtained [48].

### 4.6. Validation of RNA-Seq by Quantitative Real-Time PCR (qPCR)

Specific primers for randomly selected genes were designed using the NCBI website, with EF1a serving as the internal reference (Supplementary Table S5). RNA was extracted from samples and, after assessing their purity and concentration, double-stranded cDNA was synthesized via reverse transcription with a reagent kit, according to the manufacturer’s instructions. Quantitative real-time PCR (qPCR) was performed on a QuantStudio™ 3 Real-Time PCR System (Thermo Fisher Scientific, Sunnyvale, CA, USA). The Cq values obtained were used to calculate the fold change in gene expression using the 2^−ΔΔCT^ method [49].

## Figures and Tables

**Table 1:** Statistics of alkaline stress phenotypic data for 258 wheat accessions.

Trait	Min (cm)	Max (cm)	Mean (cm)	SD	CV (%)
RL_CK_1d	5.60	11.10	8.76	1.06	12.14
RL_T_1d	2.00	5.63	3.74	0.71	19.05
RL_CK_3d	9.33	17.55	14.09	1.40	9.93
RL_T_3d	1.95	5.95	3.80	0.74	19.58

Note: RL, root length; CK, control; T, treatment.

**Table 2:** Loci associated with root traits under control and alkaline stress conditions identified via GWAS.

Traits	SNP	Chr	Pos	−log_10_(*P*)	*R*^2^%	Effect	Model
RL-CK-1d	2A_471531993	2A	471531993	4.18	5.28	−0.54	GLM
RL-CK-3d	1B_115442787	1B	115442787	4.96	7.31	−0.67	GLM
RL-CK-3d	1B_115442787	1B	115442787	4.18	7.13	−0.66	MLM
RL-CK-3d	1B_119602852	1B	119602852	5.20	7.83	−0.69	GLM
RL-CK-3d	1B_119602852	1B	119602852	4.47	7.86	−0.69	MLM
RL-CK-3d	1B_129994772	1B	129994772	4.25	6.23	−0.61	GLM
RL-CK-3d	1B_134605692	1B	134605692	5.03	7.68	−0.67	GLM
RL-CK-3d	1B_134605692	1B	134605692	4.25	7.47	−0.66	MLM
RL-CK-3d	1B_167777201	1B	167777201	4.67	7.16	−0.65	GLM
RL-CK-3d	1B_167777201	1B	167777201	4.05	7.23	−0.65	MLM
RL-CK-3d	1B_219513011	1B	219513011	4.60	6.85	0.63	GLM
RL-CK-3d	2A_313405793	2A	313405793	4.49	15.71	−0.80	GLM
RL-CK-3d	2A_313405793	2A	313405793	4.12	16.45	−0.82	MLM
RL-CK-3d	3B_3239089	3B	3239089	4.52	6.67	−0.64	GLM
RL-CK-3d	4B_817608	4B	817608	4.12	3.89	−0.63	GLM
RL-CK-3d	4B_417309850	4B	417309850	4.03	4.01	0.41	GLM
RL-CK-3d	5B_6485240	5B	6485240	4.04	3.89	−0.42	MLM
RL-CK-3d	7B_14912720	7B	14912720	4.80	8.22	−0.70	GLM
RL-CK-3d	7B_14912720	7B	14912720	4.92	8.70	−0.72	MLM
RL-T-1d	3B_322640	3B	322640	4.45	6.34	0.33	GLM
RL-T-1d	3B_704997	3B	704997	4.56	6.54	0.34	GLM
RL-T-1d	3D_41688704	3D	41688704	4.43	4.46	−0.25	GLM
RL-T-1d	3D_63239049	3D	63239049	4.11	4.38	0.22	GLM
RL-T-1d	5B_491948869	5B	491948869	4.01	3.42	0.34	GLM
RL-T-1d	5B_557502580	5B	557502580	4.35	5.03	−0.26	GLM
RL-T-3d	3B_322640	3B	322640	4.06	5.74	0.33	GLM
RL-T-3d	3B_704997	3B	704997	4.14	5.90	0.33	GLM
RL-T/CK-1d	1A_551399390	1A	551399390	4.13	4.22	−0.02	GLM
RL-T/CK-1d	4A_481510017	4A	481510017	4.22	4.88	−0.05	MLM
RL-T/CK-1d	4A_481510017	4A	481510017	4.33	4.42	−0.05	GLM
RL-T/CK-1d	5B_557502580	5B	557502580	4.67	6.14	−0.03	MLM
RL-T/CK-1d	5B_561705149	5B	561705149	4.03	4.24	−0.02	GLM
RL-T/CK-1d	5B_557502580	5B	557502580	4.83	5.73	−0.03	GLM
RL-T/CK-3d	5B_557502580	5B	557502580	4.32	5.03	−0.02	GLM
RL-T/CK-3d	5B_557502580	5B	557502580	4.22	5.06	−0.02	MLM

Note: RL, root length; CK, control; T, treatment.

**Table 3:** Common genes identified via GWAS and RNA-seq.

Gene ID	Associated SNP	Chr	Position (bp)	Description	Genotype 182 (Tolerant)		Genotype 238 (Sensitive)	
					T1/CK	T2/CK	T1/CK	T2/CK
TraesCS1A03G0917700	1A_551399390	1A	551,591,836–551,593,360	Transcription factor RAX2; regulates axillary meristem development; contains Myb-like DNA-binding domain	4.06	6.04	3.16	5.56
TraesCS1A03G0913800	1A_551399390	1A	550,967,927–550,969,339	Beta-1,3-galactosyltransferase (PvGal synthesis protein 3); involved in glycosylation and stress response	2.10	4.17	2.05	3.42
TraesCS3D03G0225500	3D_63239049	3D	63,007,082–63,008,233	Zinc finger CCCH domain-containing protein 2 (OsDOS); regulates senescence and stress responses	2.35	2.25	2.98	3.71
TraesCS3D03G0226000	3D_63239049	3D	63,405,847–63,409,862	ATP-dependent 6-phosphofructokinase 3 (ATP-PFK 3); key enzyme in glycolysis	2.39	2.84	2.11	2.87
TraesCS5B03G0953100	5B_557502580	5B	558,417,547–558,422,277	Organic cation/carnitine transporter 7 (AtOCT7); member of sugar and other transporter family	2.21	1.90	2.52	2.37
TraesCS5B03G0961700	5B_561705149	5B	563,149,203–563,153,131	Transcription factor bHLH148 (OsbHLH148); regulates stress-responsive genes	4.51	8.10	6.57	8.62
TraesCS3D03G0227400	3D_63239049	3D	64,080,875–64,083,929	Profilin; involved in cytoskeleton organization; contains Profilin domain	0.36	0.34	0.40	0.33

Note: T1, 6 h; T2, 24 h; CK, control.

## Data Availability

The RNA-seq data has been deposited at the National Center for Biotechnology Information (NCBI) and the accession number is PRJNA1290841.

## Supplementary Materials

The following supporting information can be downloaded at: https://www.mdpi.com/article/10.3390/ijms26178659/s1.
