# Supplementary material for: Genome-Wide Association Study and Transcriptome Analysis Reveal Alkaline Stress-Responsive Genes in Bread Wheat (Triticum aestivum L.)
Source: Int J Mol Sci. 2025 Sep 5;26(17):8659. doi: 10.3390/ijms26178659 (PMC12429589; doi:10.3390/ijms26178659)
Supplement: Supplementary file 1 [file ijms-26-08659-s001.zip › Supplementary Table S5. Information on all primers used in this study.pdf]

Supplementary Table S5. Information on all primers used in this study.

Gene Name	Forward Primer (5'–3')	Reverse Primer (5'–3')
EF1a	GTGCTGTTCTCATCATCGACTC	CTCCAAGAGTGAAAGCAAGGAG
TraesCS2D03G1218200	CATCTAAAGCTTGAGGGTTTGCAG	AGAGAGAGCTTTGATAACAGGGTC
TraesCS4A03G1222600	TCGGGTCCAAGGACAACCAC	TCACGTACAGCTCCTCTGAG
TraesCS2B03G1445600	CAAGCTTTGTTGCCGATGCG	CCTCTACATTGGGCCAGAATAC
TraesCS7A03G0072700	GCCGCCTATGCAAACGACAA	ATGGCGTCACCACCGAAGAA
TraesCS2B03G1233400	TTTTACAGCCGAGAGGATCG	CCCCCGTGTTTGAAATTACG
TraesCS3A03G1126300	ACCACCGTGAACGACAAC	TCAACCCCTGGTTATACGCA
TraesCS2B03G1060800	TCGCCTAAGTCCGACAAGAA	GTATCAGGCGTATCAGCG
TraesCS2B03G1047000	CACCAGGAGGACATGCACAC	TCCTCCTCTTCGCTGACCTC
TraesCS1B03G0361800	TGCCTACTTTCTGATGATCCTG	AAAAGCTTGTACGGGGAGGA
