# Supplementary figures and images for: Genome-Wide Association Study and Transcriptome Analysis Reveal Alkaline Stress-Responsive Genes in Bread Wheat (Triticum aestivum L.)
Source: Int J Mol Sci. 2025 Sep 5;26(17):8659. doi: 10.3390/ijms26178659 (PMC12429589; doi:10.3390/ijms26178659)

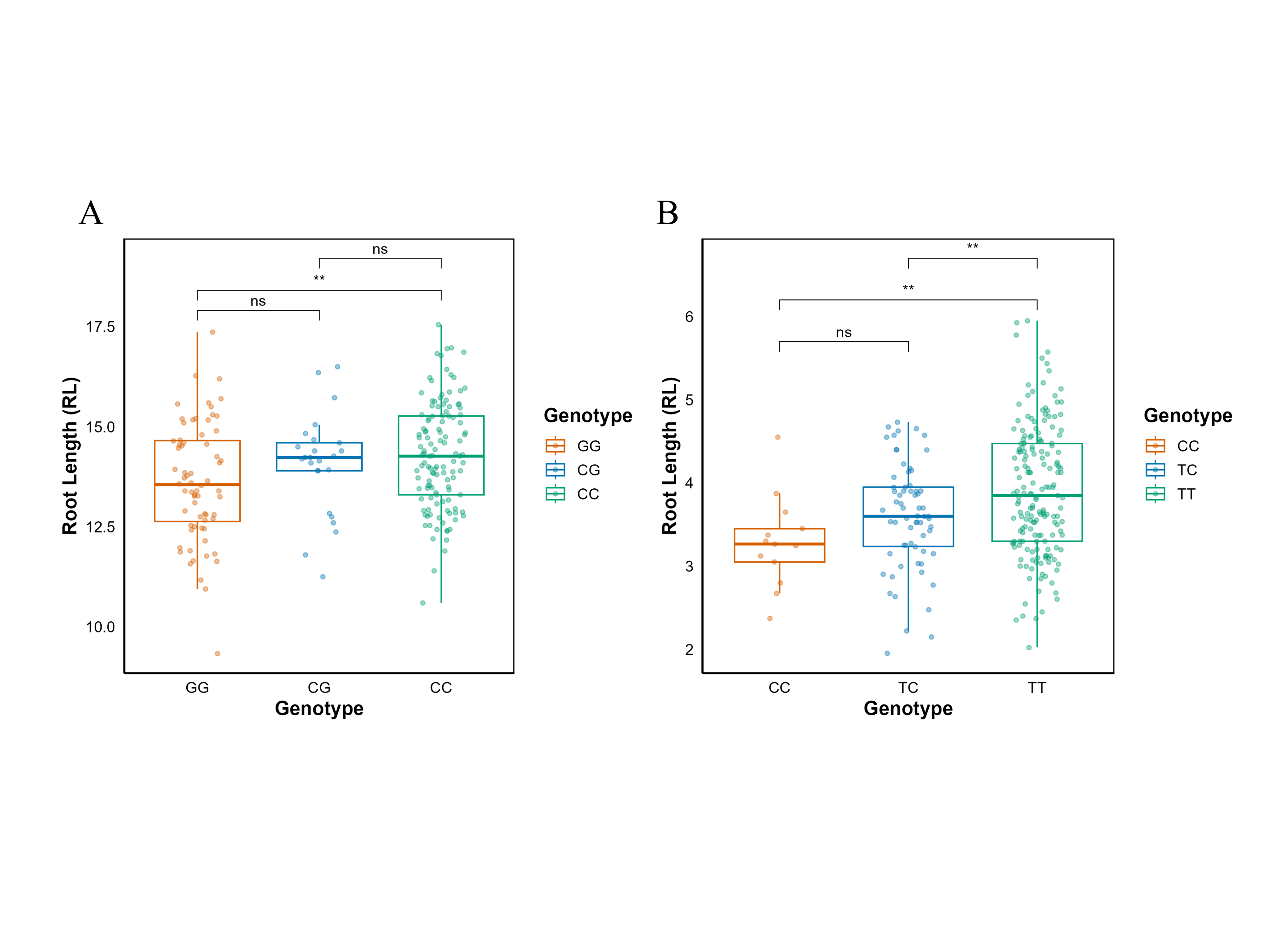

Supplement: Supplementary file 1 [file ijms-26-08659-s001.zip › Supplementary Figure S1. Boxplots showing the association between genotype and root length (RL) at two significant SNP loci.tif]
